# Cytokine removal in human septic shock: Where are we and where are we going?

**DOI:** 10.1186/s13613-019-0530-y

**Published:** 2019-05-14

**Authors:** Patrick M. Honore, Eric Hoste, Zsolt Molnár, Rita Jacobs, Olivier Joannes-Boyau, Manu L. N. G. Malbrain, Lui G. Forni

**Affiliations:** 1 Intensive Care Department, CHU Brugmann University Hospital, 4, Place Arthur Van Gehuchtenplein, 1020 Brussels, Belgium; 2 Intensive Care Department, Ghent University Hospital, Ghent, Belgium; 3 Department of Anaesthesiology and Intensive Therapy, Faculty of Medicine, University of Szeged, Szeged, Hungary; 4 Intensive Care Department, University Hospital Brussels (UZB), Jette, Belgium; 5 Département d’Anesthésie-Réanimation SUD, CHU Bordeaux, 33000 Bordeaux, France; 6 Faculty of Medicine and Pharmacy, Vrije Universiteit Brussel (VUB), Brussels, Belgium; 7 Department of Critical Care, Royal Surrey County Hospital, NHS Foundation Trust, Guildford, UK; 8 Department of Clinical and Experimental Medicine, Faculty of Health Sciences, University of Surrey, Guildford, UK

**Keywords:** Blood purification, Cytokines, Sepsis, Septic shock, Haemoperfusion, Cytosorb, Sorbents, Cartridges, Immune modulation, DAMPS, PAMPS

## Abstract

Although improving, the mortality from septic shock still remains high despite increased international awareness. As a consequence, much effort has focused on alternative treatment strategies in an effort to improve outcomes. The application of blood purification therapies to improve immune homeostasis has been suggested as one such method, but these approaches, such as high-volume continuous haemofiltration or cytokine and/or endotoxin removal, have enjoyed little success to date. More recently, the use of sorbent technologies has attracted much attention. These adsorbers are highly effective at removing inflammatory mediators, in particular, cytokines, from the bloodstream. This narrative review is the executive summary of meetings held throughout the 6th International Fluid Academy Days in Antwerp, Belgium (Nov 23–25, 2017), focusing on the current understanding regarding the use of such adsorbers in humans with septic shock. We followed a modified Delphi approach involving a combination of evidence appraisal together with expert opinion in order to achieve recommendations for practice and, importantly, future research.

## Introduction

### The pathogenesis of sepsis: blood as “biopsy” of tissue inflammation

Septic shock continues to have significant mortality [1]. The underlying pathophysiology is complex with both pathogenic and host factors (PAMPs (pathogen-associated molecular patterns) and DAMPs (damage-associated molecular patterns)) playing a significant role in the development and subsequent outcome [2]. However, the heterogeneity of septic shock prevents adequate characterization of patients and may hinder subsequent clinical intervention(s). Sepsis and septic shock affect anywhere between 100 to 1000 per 100,000 person-years and 19 per 100,000 person-years depending on the cohort studied [3–6], with reported mortality rates ranging between 20 to 50% [7–11]. Moreover, the reported incidence is increasing, although this may be attributed to reporting bias in so-called claims-based databases, as data analysis on electronic health records cannot confirm this trend [12–15]. Therefore, it is of no surprise that the treatment of sepsis has become a major global health issue. Indeed, in the USA during 2011 sepsis accounted for just over 5% of total hospital costs corresponding to $20 billion dollars [16].

### The place for blood purification

Patients with sepsis are often treated in areas of intensive care given that close monitoring and intense therapeutic support are needed [17]. Early treatment includes the use of timely, appropriate antibiotics, intravenous fluids, oxygen therapy as well as vasopressor and inotropic support where needed. Other additional treatments including extracorporeal or so-called blood purification techniques (BPT) have also been tried [18]. These techniques include (among others): haemofiltration, haemoperfusion, intermittent or continuous high-volume haemofiltration (HVHF), plasmapheresis or adsorption. The rationale behind such an approach is to achieve “immune homeostasis” which theoretically reduces the potential damage caused by dysregulation of the host response to infection. This may be heralded by a profound rise in inflammatory mediators including cytokines which contribute to the dramatic systemic effects of sepsis, mainly in septic shock [9, 19]. The recently updated Sepsis 3.0 consensus definitions state that sepsis is an infection accompanied by life-threatening organ dysfunction caused by a dysregulated host response [20]. Given the pivotal role of cytokine production in sepsis, it follows that removal of these substances, through such BPT, may attenuate the response particularly in the early phase of sepsis [21]. Several hypotheses have been proposed as to the potential mechanisms underpinning potential benefit. These include cytotoxic theories including the **peak concentration hypothesis** whereby all inflammatory mediators are removed at a given rate, dependent on the BPT used and assuming they are filtered [21–24]. Alternatively, the **cytokinetic theory** proposes that cytokines are removed, thereby creating a cytokine gradient between the bloodstream and tissues allowing leucocyte-enhanced trafficking [25]. In the same line, cytokine levels can also be seen as communicating messengers to talk to cells, recruit some, depress others and reduce cell metabolism for others [22]. Despite early promise, no multicentre randomized controlled studies have demonstrated a survival benefit including the use of HVHF where higher flows may lead to increased cytokine removal were tried (**the cytotoxic threshold immune modulation hypothesis**) [26–28]. Other extracorporeal blood purification therapies also have failed with significant outcome data lacking with no treatment demonstrating a translatable survival benefit in any randomized controlled study [29–31]. This somewhat too simplistic view takes into account several new concepts. Indeed, blood level of mediators (more than cytokines) implies the saturation of the interstitial and cellular compartments to be present in the blood. It is the so-called **tip of the iceberg theory** [31]. This theory is very similar to the “threshold immune modulation theory” [23]. In addition, plasma mediator levels depend on several factors: the intensity of production, the number of cell receptors availability, the clearance of such mediators, the affinity of the receptors for such mediators. As an example, the interleukin (IL)-6 receptor is an agonist one, which differs from tumour necrosis factor (TNF)-soluble receptors and IL-1 receptors that are inhibitory; the consequence is that a low IL-6 level with a high level of receptor induces more cellular response than high level of IL-6 with a low level of receptors [22]. This very important issue is showing that an IL-6 level alone may not be very predictive of the future response of the organism. DAMPs are also playing a key role and especially the endosomal DAMPs that are eliminated via the BPT and are capable of inducing cellular damage and apoptosis [32]. This may explain the initial “positive” observational trials with HVHF. The reported improvement in hemodynamic status associated with or not with lactate reduction might mainly be the result of the fluid replacement therapy made with large volumes of crystalloids containing high buffer concentrations. As a consequence, the induced pH increase might have changed the affinity for catecholamines to their receptors improving hypotension [28–30].

### Impact on mortality

Extracorporeal haemoperfusion with Polymyxin B (PMX-HP) has shown improvement in organ dysfunction and a survival benefit in small studies [33] including a small randomized trial [34], while larger trials failed to confirm these findings [35, 36]. Evaluating the use of PMX-HP in a randomized controlled trial of adults treated for endotoxemia and septic shock (the EUPHRATES study) included patients with persistent septic shock despite adequate fluid resuscitation and vasopressor treatment [37]. An endotoxin activity assay (EAA) was applied. No mortality difference was observed in the “per protocol population” (*n* =  244, multiple organ dysfunction score (MODS) > 9, EAA ≥ 0.6). However, among the patients in refractory shock with MODS of more than 9 and an EAA between 0.6 and 0.9, a significant 10.7% reduction in 28-day mortality in a post hoc analysis was recognized after receiving two sessions of PMX-B-HP [38]. Post-hoc analysis revealed a significant mortality reduction when the EAA was limited to less than 0.9, which may suggest an upper limit to a pre-treatment endotoxin burden under these treatment conditions [38]. A recent meta-analysis of 17 trials demonstrated that PMX-B-HP treatment may reduce mortality in patients with severe sepsis and septic shock. The included studies were stratified into three groups based on the mortality rates of the conventional treatment group: low-risk group (mortality rate < 0.3), intermediate-risk group (0.3–0.6) and high-risk group (> 0.6). Risk ratios with 95% confidence intervals (CI) for the mortality-stratified analysis between the PMX-HP and conventional treatment groups were calculated and presented as summary statistics. This a posteriori classification can be seen as a major caveat of this meta-analysis. Also mortality rate does not reflect cytokine concentrations and load and therefore is not a predictor of response to cytokine removal. Disease severity subgroup meta-analysis revealed a significant risk reduction in overall mortality in the intermediate-risk (mortality rate 0.3–0.6) and high-risk (mortality rate > 0.6) groups, but not in the low-risk (mortality rate < 0.3) group reinforcing the view that rigorous patient selection is crucial for treatment success [39].

Lastly, two editorials set the pace by stating that while blood purification in sepsis remains a valid approach, the use of adsorbers and their current efficacy of endotoxin/cytokine elimination cannot be recommended to reduce the mortality in absence of positive randomized controlled trials (RCTs) [40, 41].

### Cytokine storm and capillary leak

Capillary leak represents the maladaptive and undesirable movement of fluid and electrolytes with or without protein into the interstitium that generates anasarca and end-organ oedema potentiating organ dysfunction and or failure [42]. The global increased permeability syndrome (GIPS) defined as a positive cumulative fluid balance and new onset organ dysfunction/failure is described in patients with persistent systemic inflammation resulting in continuing transcapillary albumin leakage. GIPS may represent the third phase in a continuum after the initial cytokine storm and ischaemia–reperfusion injury and could be a potential indication to start BPT [43].

### Finding the answers

The focus of this consensus meeting was to determine whether a reduction in cytokine levels is possible through the use of sorbent technology by use of the CytoSorb^®^ (Cytosorbents, Corporation, New Jersey, USA) device. This consists of a single-use haemoadsorption cartridge which can be used with standard blood pumps, such as those found on RRT machines or through a haemoperfusion device [44–46]. The CytoSorb cartridge is the only currently available CE-marked device shown to consistently lower excessive cytokines in severe sepsis. The cartridge is filled with sorbent beads made from a porous polymer that adsorbs and capture cytokines as blood passes through the device. This process is concentration dependent, and so the higher the levels of cytokines in the blood, the faster the levels are reduced. Although CytoSorb therapy has been described for other indications, including intoxications, rhabdomyolysis, hyperbilirubinemia, etc., we chose to concentrate on human studies in sepsis and septic shock.

## Consensus process

This consensus meeting took place during the 6th International Fluid Academy Day (IFAD) held in Antwerp, Belgium, at the end of November (November 23–26, 2017). We followed a modified Delphi approach involving a combination of evidence appraisal together with expert opinion in order to achieve recommendations for practice and future research. A panel of clinicians representing intensive care, anaesthesia and critical care nephrology from Europe convened to discuss the issues related to cytokine haemoadsorption using the CytoSorb^®^ or other devices in humans with sepsis and septic shock. The participants were tasked with summarizing the literature in humans to date, highlighting knowledge gaps and recommending areas of potential research. Reviews of the literature were performed prior to the meeting with a presentation to the participants and consensus was reached. MEDLINE^®^ and PubMed searches were performed using the search terms “cytosorb”, “haemofiltration”, “haemoadsorption”, “haemoperfusion” and “‘sepsis OR septic shock OR critical care OR critical illness’”. The reference lists of identified papers were screened to identify other relevant papers. Where necessary, further discussion was performed after the meeting. Although representatives from industry were present, they did not contribute to the discussion unless specifically questioned and did not play a role in the manuscript preparation. This paper serves as the final Executive Summary of the meeting.

## Pathophysiology

**Fig. 1:**
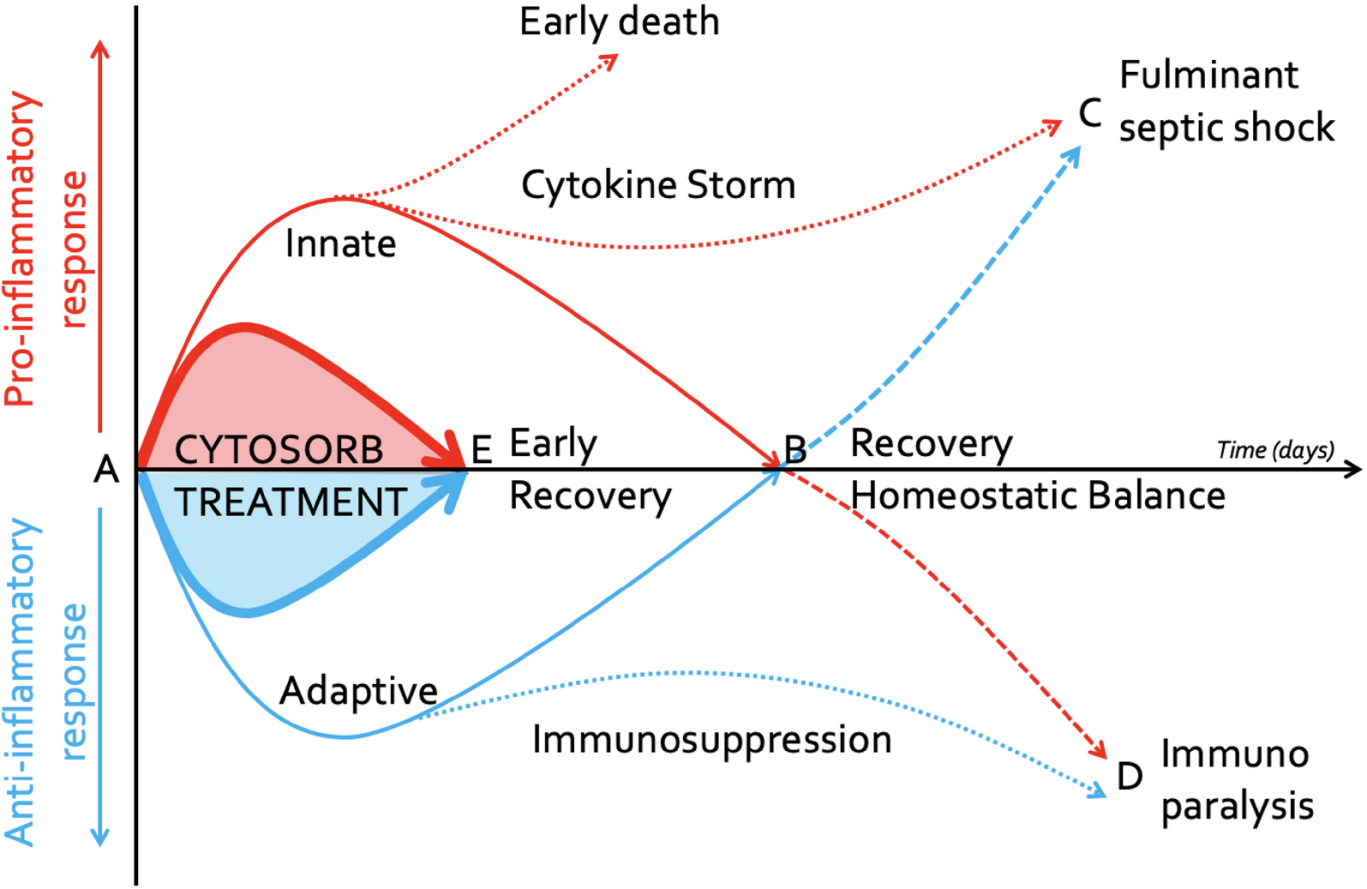
Cytokine response after sepsis. Normal and abnormal immune response after an (infectious) insult (A). Recovery with regaining of the homeostatic balance occurs when pro-inflammatory (solid red line) and anti-inflammatory (solid blue line) mediators (B) return back to baseline levels. Early death or fulminant septic shock (C) can occur following early increased innate pro-inflammatory response (cytokine storm, dotted red line) or after initial adaptive immunosuppression (dashed blue line). Immunoparalysis (D) can occur following early increased adaptive anti-inflammatory response (immunosuppression, dotted blue line) or after initial pro-inflammatory response (dashed red line). Haemoadsorption with Cytosorb^®^ may attenuate the initial pro- (bold red line) and anti-inflammatory (bold blue line) response resulting in early recovery (E)

**Fig. 2:**
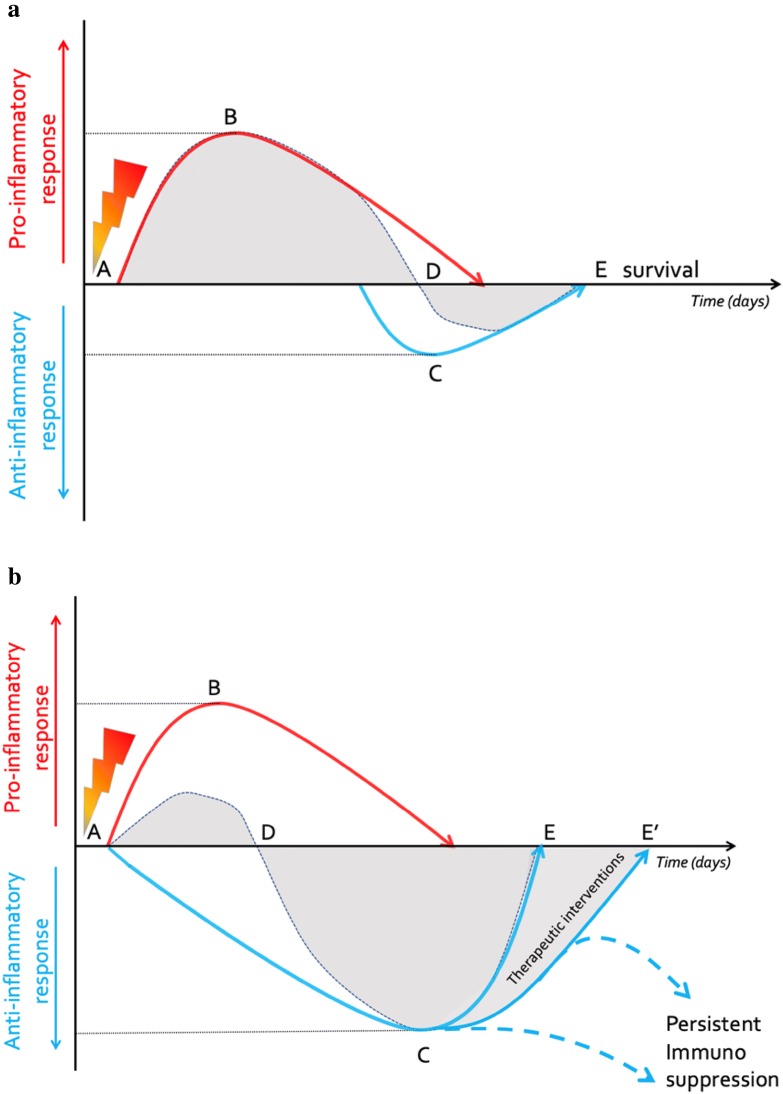
Balance between the pro- and anti-inflammatory mediators. **a** Following an initial (infectious) insult (A), normally after correct (antibiotic) treatment the antagonistic forces of pro-inflammation (B), and anti-inflammation (C) regain balance (grey area shows net effects) maintaining healthy homeostasis (D), that will lead to recovery and survival (E). Adapted from Pfortmueller et al. with permission (Open Access CC BY Licence 4.0) Intensive Care Medicine Experimental (2017) 5:49 10.1186/s40635-017-0163-0. **b** Following an (infectious) insult (A), during a dysregulated host response the pro-inflammatory (B) forces initially overwhelm anti-inflammation (C) resulting in an imbalance (D), followed by immunosuppression and increased anti-inflammatory mediators (grey area shows net effects). With different therapeutic interventions recovery (E) can be obtained or delayed (E’) or the patient can evolve into a state of persistent immunosupression or paralysis. Adapted from Pfortmueller et al. with permission (Open Access CC BY Licence 4.0) Intensive Care Medicine Experimental (2017) 5:49 10.1186/s40635-017-0163-0

As discussed, the inflammatory state associated with sepsis leads to release into the circulation of many pro-inflammatory mediators leading to deleterious systemic effects [44]. Although this effect may in part be modified through anti-inflammatory mediators, sustained effects may lead to relative immunoparesis [47, 48]. This concept is important given the overused simplistic view that sepsis is associated with a significant uncontrolled release of pro-inflammatory mediators and simple modulation of these would translate into improved patient outcomes [21]. Hence the desire for a more specific targeted therapy, rather than just adoption of “standard” continuous renal replacement techniques as a profound pro-inflammatory response, may lead to dysregulation of the anti-inflammatory pathways, and the aim of immune homeostasis may be thwarted by a worsening of the host response (Figs. 1, 2). Indeed, such dysregulation can be seen in patients with sepsis who transition to a late anti-inflammatory phenotype referred to as sepsis-associated immunosuppression (SAI) [49–51]. This process involves the reduction in the production of the inflammatory mediators together with direct effects on antigen-presenting activity. In turn, this may lead to enhanced immune tolerance with changes demonstrated in circulating immune cells and within tissues [52]. Following injury or infection, there is activation of humoral factors such as complement that then trigger the antigen-presenting cells (APCs) that release a host of mediators including cytokines. In turn, these attract and, as a consequence, activate more APCs and neutrophils. Migration to draining lymph nodes stimulates further adaptive immune activity. These processes are followed by the anti-inflammatory response whereby there is a diminution of the host response as a consequence of anti-inflammatory cytokines such as IL-10 being produced. In the critically ill, this may lead to immunosuppression as described [47, 48]. Cytokines play a pivotal role in the progression of the sepsis response. In early sepsis TNF-α, a pro-inflammatory cytokine released by monocytes and macrophages, is a marker of early sepsis and enhances the adaptive immune response [53]. Several studies have demonstrated an association between mortality and elevated TNF-α although the utility of TNF-α as a predictor of mortality has been questioned [54]. IL-6, predominantly produced by monocytes and macrophages, induces T-cell activation and B cell proliferation and stimulates the acute phase response all leading to augmentation of the immune response [55]. Circulatory levels rise rapidly after infection with peak levels approaching 2 h after insult with increased levels associated with poorer outcomes. However, cytokines may be derived from non-immune cells and have variable clearance rates and as such may not reflect immune cell functionality in all cases [56]. Furthermore, IL-6 has a relatively fast induction coupled with a short half-life, and these characteristics make it ideally suited for patient monitoring although to date most laboratories do not offer routine assay. With regard to anti-inflammatory cytokines, IL-10 is the most studied and has similar kinetics to IL-6. However, unlike IL-6, Il-10 induces antigen tolerance enhancing SAI and may predict mortality [57]. Obviously, considering IL-6 as a potential target remains interesting, but the profile of IL-6 kinetics in critically ill patients could be heterogeneous and explained by several factors. Indeed, in the abdomix study, some patients were with and without high levels of IL-6 also in the control arm [35, 36].

## Rationale for cytokine removal

### Rationale

**Fig. 3:**
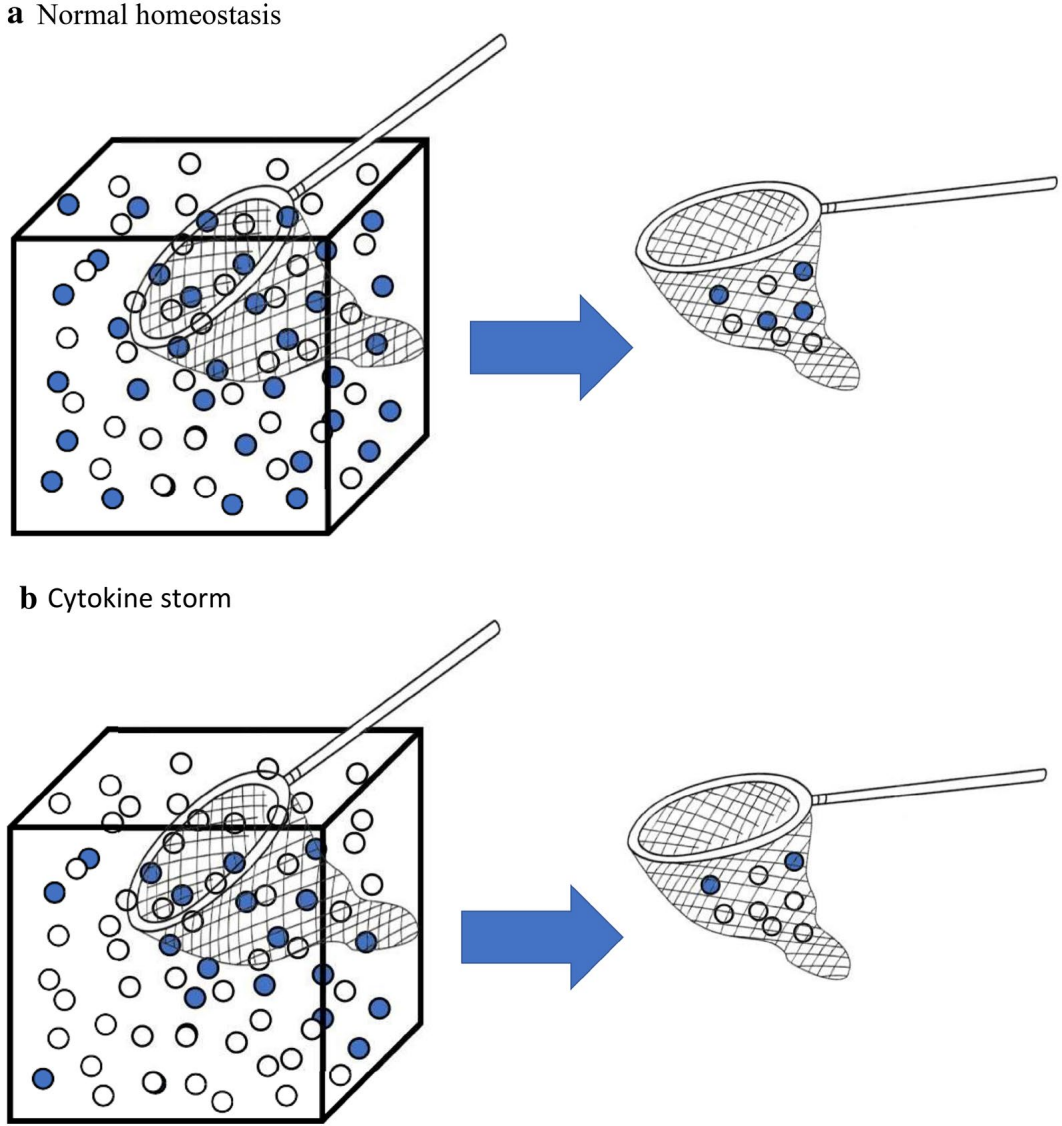
The rationale of bulk removal of cytokines during cytokine storm. When homeostasis is normal, the pro-inflammatory (open circles), and anti-inflammatory (closed circles) mediators are in balance, molecules are present evenly as demonstrated in the first panel of the figure (**a)**. When molecules are adsorbed in this scenario, the removal rate of both should be similar. However, if one component is in abundance—which is the case during cytokine storm (**b**), then the removal rate should be proportionally higher from the mediator that is present in large numbers. This demonstrates the rationale why bulk removal of cytokines during cytokine storm may help to regain homeostatic balance

The enhanced inflammatory response seen in septic shock is associated with a high mortality [57, 58] correlated with the production of pro- and anti-inflammatory mediators [57] rather than disequilibrium between pro- and anti-inflammatory mediators [59] (Fig. 3). This has stimulated much effort towards potential attenuation of this response particularly as early studies suggested that continuous veno-venous haemofiltration (CVVH) may reduce cytokine levels [60]. However, as discussed, these early observations have not translated into clinical benefit. Meta-analysis suggests that the only potentially effective BPT for the treatment of sepsis are plasma exchange or haemoadsorption particularly with Polymyxin B [2, 39]. Although initial results using Polymyxin B haemoperfusion showed promise [31], this has not been borne out by subsequent randomized clinical trials [32]. Given the remit of sorbent technologies, we chose to focus on the use of CytoSorb as a haemoadsorption device. The adsorber has a surface of about 45,000 m^2^ compared to a conventional hemofilter with a surface of 1–1.5 m^2^ with a molecular cutoff of about 60 kDa removing cytokines as well as other toxins and drugs. As a consequence, CytoSorb does not adsorb endotoxin which has a molecular weight of 100 kDa. CytoSorb is saturable regarding adsorption in the clinical setting (mostly after 8 h) as evidenced by an rebound increase in the dose of vasopressors which can be tapered when changing the CytoSorb. So far, in vitro studies have not been evaluated regarding the adsorption–saturation process of adsorption. This could be an interesting area for future research. Multiple pre-clinical studies using animal models of sepsis have demonstrated reductions in various circulating cytokines and chemokines, reduced organ injury, and improved survival [61–64].

### Impact on different cytokines

In a feasibility study, the removal of cytokines was confirmed with cytokine extraction rates approaching 30% [65]. In this study, plasma concentrations of both IL-6 and TNF-α, but not IL-10, were significantly reduced after the first hour of therapy [65]. Overall removal was greatest for IL-6, 28% (*p* = 0.006), and least for tumour necrosis factor, 8.5% (*p* = 0.13). However, plasma concentrations for all three cytokines increased over time and were above baseline by the end of the intervention (4 h). In a more recent study, the group of Antoine Schneider [66] conducted a single-centre pilot randomized controlled trial in 30 patients undergoing elective cardiac surgery and deemed at risk of complications. Patients were randomly allocated to either standard of care (*n* = 15) or CytoSorb^®^ haemoadsorption (*n* = 15) during cardiopulmonary bypass (CPB). The primary outcome was the difference between the two groups in cytokines levels (IL-1a, IL-1b, IL-2, IL-4, IL-5, IL-6, IL-10, TNF-α, interferon gamma (IFN-γ), monocyte chemotactic protein-1 (MCP-1) measured at anaesthesia induction, at the end of CPB, sand 6 and 24 h post-CPB initiation. However, the intervention was associated neither with a decrease in pro- or anti-inflammatory cytokine levels nor with any improvement in relevant clinical outcomes [66]. These results are conflicting, and this is obviously a major concern regarding this new technique. So, altogether, no study using CytoSorb has shown a sustained plasma concentration reduction lasting during the whole treatment. Given the possibility that this technique may hold promise, we reviewed available data with the aim of producing current consensus statements and directing further research [67–69].

## Does the use of CytoSorb haemoadsorption therapy in patients with sepsis or septic shock demonstrate any clinical benefit?

Although there is a significant body of experimental evidence supporting the potential use of haemoadsorption with CytoSorb in septic shock, there is far less evidence from human studies. There are over 100 case studies describing the use of CytoSorb in many clinical scenarios, and in general, the treatment is well tolerated, but clinical studies in general are small case series [70–72]. The largest study to date, a randomized controlled, open-label study enrolled patients from 10 German study sites over the period 2008–2011 [73, 74]. A total of 582 patients were screened for this study of which 100 mechanically ventilated patients with severe sepsis or septic shock together with acute lung injury or acute respiratory distress syndrome were recruited. Final analysis was limited to 97. Remarkably, no data regarding hemodynamic indices such as cardiac index or systemic vascular resistance was recorded. Patients were randomized initially on a 1:1 basis using sealed envelopes, but due to suspected irregularities, this system was changed after 32 patients were recruited to an electronic system using a block length of 6. The treatment arm received 6 h of CytoSorb haemoperfusion per day for up to 7 days compared to standard care. The primary endpoint was a reduction in “normalized” rather than absolute IL-6 levels with post-treatment IL-6 levels divided by the individual patients’ baseline IL-6 concentration. Unfortunately, in 22 patients, no valid primary endpoint was available leaving 75 patients in the final cohort. Secondary endpoints included a host of other cytokines, duration of mechanical ventilation, 28-day mortality and the MOD score. The primary endpoint of this study was negative. The use of haemoadsorption was not associated with a reduction in IL-6 levels. After adjusting for comorbidities (age, gender, and RRT), there was no association between treatment with haemoadsorption and mortality or indeed any of the other secondary endpoints. What was noted was that there were a larger number of clinically significant patients needing RRT in the treatment group (31.9% vs 16.3%). Although the application of the CytoSorb cartridge did result in detectable IL-6 elimination (5-18%) throughout the 6-h period, this did not translate to an overall reduction compared to control. This may appear incongruous as the effects of treatment on IL-6 between groups were measured at day 2 and not during the time periods employed to assess IL-6 removal (15, 60, 180 and 360 min, respectively) [73, 74].

Although this can be regarded as the first randomized controlled trial (RCT) employing the CytoSorb technique in sepsis, the results do not provide any evidence that treatment resulted in a clinically relevant endpoint. The study was powered for safety and efficacy of IL-6 removal. The patient population included was also less sick then in case series and in a recent registry [75], suggesting that since 2008, CytoSorb has been used in a sicker population. Similarly, a prospective randomized control trial in patients who underwent cardiac surgery also found no significant impact on cytokine concentrations although this study was not performed in sepsis [76]. In a recent propensity-matched retrospective study on 32 patients, the influence of intraoperative cytokine adsorption on the perioperative vasoplegia, inflammatory response and outcome during orthotopic heart transplantation was investigated [77]. Intraoperative CytoSorb treatment was associated with reduced vasopressor demand and less frequent RRT with no difference in length of mechanical ventilation and ICU stay [78]. Other case series in sepsis patients do point to a reduction in vasopressor dose, which may be considered a relevant endpoint, although these are not randomized studies [68–70]. The third interim analysis of the CytoSorb Registry [75] of 135 patients with septic shock and an acute physiology and chronic health evaluation II (APACHE-II) predicted mortality of 78% had an observed mortality of 65% with a marked reduction in both PCT and IL-6 levels after 24 h [75]. Such an high predicted mortality (78%) can be explained by various reasons. First, it was recommended to only use Cytosorb haemoadsorption in patients with an APACHE-II score above 25. Second, clinicians were only starting the treatment as a salvage therapy, late in the course of the disease. Obviously, with a registry, you cannot control the eligibility criteria. Recently, the first randomized clinical trial on CytoSorb as a stand-alone (i.e.: without CRRT) haemoperfusion treatment in patients with septic shock, the ACESS trial (Adsorption of Cytokines Early in Septic Shock) was published [78, 79]. This was a proof of concept pilot study on 20 medical patients randomized into a CytoSorb and a standard treatment group, with cytokine adsorption initiated within the first 24 h after the onset of septic shock. The treatment proved to be safe and resulted in a significant reduction in norepinephrine requirement in the CytoSorb group as compared to controls: T0 = 0.54[IQR: 0.20–1.22], T48 = 0.16[IQR: 0.07–0.48], *p* = 0.016; Controls: T0 = 0.43[IQR: 0.19–0.64], T48 = 0.25[IQR: 0.08–0.65] µg/kg/min. They also observed a significant reduction in procalcitonin (PCT): CytoSorb: T0 median = 20.6[IQR: 6.5–144.5], T48 = 5.6[1.9–54.4], *p* = 0.004; Control: T0 = 13.2[7.6–47.8], T48 = 9.2[3.8–44.2] ng/mL [78, 79]. The promising results of this pilot study may serve as the rationale of further large randomized clinical trials, but at present, there is a lack of robust evidence to support recommendations.

## Results of the consensus meeting

To date, there is not enough data to provide any kind of evidence-based recommendations for the use of sorbent technologies, but the following points were considered by the panel as most important unanswered questions which may aid further recommendations.

### Point-1: Which patient would benefit the most from cytokine removal?

The aim of any treatment is to select only patients who have a high probability of benefit from the intervention. To date, most of the case series included patients with a high predicted mortality in keeping with septic shock although few have included cytokine estimation [64, 71]. Where recorded, there is significant variation in IL-6 levels, and therefore, one may conclude that some patients were treated where benefit would be negligible if indeed [IL-6] levels are predictive of response to therapy. Therefore, any future studies should attempt to enrich the study population through cytokine measurement or another marker of potential therapeutic response such as PCT [70]. A threshold PCT concentration may be included as an inclusion factor in future studies. Alternatively, patient selection may concentrate on those individuals who require high doses of vasopressor agents to maintain adequate organ perfusion as a reduction in such drugs is a meaningful clinical outcome which may translate into other benefits [78, 79].

### Point-2: When to start cytokine removal therapy in sepsis?

The timing of therapy remains an issue in many aspects of critical care medicine not least those involving extracorporeal therapies. Several of the case series report a less favourable outcome in those individuals who commenced treatment more than 24 h after diagnosis [70]. Data of the ACESS trial also support the concept of starting treatment within 24 h [78, 79]. It follows that any recommendation regarding timing of therapy will be dependent on the patient population and how prospective individuals are selected. Where the primary endpoint is a reduction in vasopressor, then timing will be relatively easy to define, whereas cytokine levels may be less clear especially given the differences in kinetics. Certainly, the rationale for any of the blood purification therapies would point to commencing treatment early in the disease process in order to maximize benefit. Maybe starting therapy directly in operating room during surgery for peritonitis patients with already hemodynamic impairment and catecholamine requirement should be considered for future studies. However, timing to intervene might be even more complex especially when sepsis-induced immunodepression is taken in account. This depression is essential to limit the consequences on the host tissues of the host response related to infection (i.e. maintain the inflammation–repair–healing cycle). Characterization of this immunodepression might be one of the best indicators to decide when to use adsorber technique to reduce inflammation, namely when the downregulation is modest or absent [22].

### Point-3: How long should cytokine removal therapy last and how long should it be continued?

The optimum length of treatment is still undecided. Moreover, the length of time of initial therapy is also unclear. Schadler et al. treated patients for 6 h a day for 7 days [73, 74], whereas other reported studies either did not describe the treatment period or used the cartridge for 24 h [70, 71]. No evidence to date exists for the appropriate duration of adsorption therapy or indeed the initial treatment period. Again, this may be directed in future studies by the use of IL-6 or rather PCT as a surrogate for total cytokine removal and reduction in systemic levels coupled with clinical improvement may be a logical place to start regarding treatment times. Also, it is unknown whether the initial therapy should be for 12 or 24 h. Again, a pragmatic approach may be directed by cytokine estimation. Again, the study by Schadler [73, 74] may provide some answers. Although the 6-h treatment did not result in an overall reduction in IL-6 compared to controls over a 24-h period, there was increased IL-6 elimination with the use of haemoadsorption [73, 74]. It follows that if the aim of treatment is to reduce cytokine levels, then a prolonged treatment period should be associated with a reduction in systemic levels. However, this removal in cytokines may be attenuated by cytokine shift from the interstitium into the blood compartment thereby negating any overall effect [22, 68, 69]. As a consequence, pharmacokinetic studies on cytokine clearance would be extremely useful to conduct in the future to better delineate the therapy duration of hemadsorption in relation to benefits for the patient with a cytokine storm.

### Point-4: Which patient population should be studied in the future?

Future studies should aim to include as homogeneous cohorts of septic shock patients, especially those with very high vasopressor needs. Another interesting population to study may be patients with acute respiratory distress syndrome (ARDS). Mortality rate of ARDS still remains high between 30 and 50% [80]. ARDS is a heterogeneous syndrome, characterized by increased pulmonary capillary permeability [81]. It is the accumulation of protein-rich fluid inside the alveoli that triggers damage to the capillary endothelium and alveolar epithelium leading to release of cytokines, producing diffuse alveolar damage [82]. Extracorporeal membrane oxygenation (ECMO) may serve as a salvage therapy with the CESAR trial that have led to an enhanced use of this therapy [83]. Several case reports mention the positive effect of incorporation of CysoSorb therapy for respiratory failure due to ARDS, using ECMO and CytoSorb therapy. The use of CytoSorb appeared to result in rapid resolution of neutropenia, reversal of toxic shock and rapid weaning of the high-dose vasopressor infusions and a significant reduction in the levels of circulating inflammatory mediators [84–88].

### Point-5: What severity of sepsis would be the most appropriate to include in a study looking at cytokine removal therapy in patients with sepsis?

The intervention is more likely to benefit patients with high severity of illness (e.g. APACHE-II greater than 25) [75]. Refractory shock indicated by high doses of vasopressor support and early multiple organ failure with some evidence of a cytokine storm (i.e.: high PCT) should be preferred [70, 71, 79].

### Point-6: Which biomarker should be the most appropriate to include in a study looking at cytokine removal therapy in patients with septic shock?

It is uncertain whether cytokine concentrations can identify the ideal patient for treatment. Detecting persistent ‘cytokine storm’—despite adequate resuscitation and source control—could be an alarming signal that cytokine removal may be beneficial. PCT as one of the most studied inflammatory biomarker in sepsis could be added as a biomarker to determine in which patient to start therapy [87, 88]. It is difficult to determine the absolute cutoff values above which treatment may be indicated, but there is some evidence, that PCT kinetics, i.e. failure of PCT to decline or rapidly increasing levels may help to identify persisting cytokine storm [89]. It is indeed the reality that the molecular weight of PCT is about 13 kDa and PCT might be removed by the adsorbent. Therefore, PCT is a good biomarker to decide when to start but not to follow the response to therapy nor to decide when to stop. IL-6 may also be a promising biomarker, but at present, little is known about IL-6 kinetics, and its response to appropriate or inappropriate treatment, which needs further research. Nevertheless, the biomarkers to characterize inflammatory status cannot be summarized by limited to IL-6 associated and PCT: we also do need to obtain information on cellular function (immune and tissular) [22].

### Point-7: Future research recommendations

Based on the above, future studies must define the population in terms of physiological parameters (APACHE-II) or SOFA (Sequential Organ Failure Assessment) scores [90], vasopressor requirement, etc.), and there should be biomarker enrichment of patient selection probably using markers such as IL-6 or PCT levels. Secondly, clinically relevant endpoints should be selected. In the first instance, this may well be vasopressor dose under strict protocol guidance and adherence. In all cases, invasive hemodynamic monitoring including cardiac output and derived variables must be performed and data recorded, in order to have a better understanding on the pathophysiological effects of the therapy. With regard to treatment length, this should be guided by response in terms of other therapies as well as biomarker reduction if possible.

Regarding study endpoints, the consensus panel agreed that other endpoints rather than mortality should be chosen in the future to test the actual effect of this therapy. Monitoring organ dysfunction with measures like the SOFA score may be a better option, in order to understand the physiological effects of CytoSorb treatment. Furthermore, a hemodynamic primary endpoint such as the change in vasopressor need could also be applied, with mortality, ICU length of stay, length of mechanical ventilation, dialysis dependence, etc., as secondary endpoints. Finally, potential side effects should be also be evaluated like the removal of antibiotics, and especially lipophilic antibiotics ones [91].

## Conclusions

To date, there is a paucity of data surrounding the use of haemoadsorption therapies in the treatment of septic shock. Although the consensus statements may appear somewhat uncertain, they reflect the current state of our understanding. Nevertheless, we hope that the current consensus paper also provides a framework for areas that need to be addressed in future work rather than continued reliance on single-centre case series.

For the time being, clinical results with the use of cytokine adsorbent therapies are scarce and rather disappointing. More studies should be performed to have a precise idea of adsorption properties (kinetics, saturability, potential mediator release, drug removal…) of the adsorbent. Plasma cytokine levels before and after treatment of various cytokines should be provided to clearly demonstrate the adsorptive properties. Only through well-conducted randomized controlled studies with appropriate patient selection criteria and endpoints of physiological relevance will we know whether haemoadsorption techniques are a future therapy for sepsis.

## Data Availability

Not applicable.

## Abbreviations

APACHE-II: acute physiology and chronic health evaluation II
ARDS: acute respiratory distress syndrome
APCs: antigen-presenting cells
BPT: blood purification techniques
CPB: cardiopulmonary bypass
CRRT: continuous renal replacement therapies
CVVH: continuous veno-venous haemofiltration
CMT: cytokine modulatory techniques
CHT: CytoSorb^®^, cytokine haemoadsorption techniques
DAMPs: damage-associated molecular patterns
EAA: endotoxin activity assay
ECMO: extracorporeal membrane oxygenation
GIPS: global increased permeability syndrome
HVHF: high-volume haemofiltration
ICU: intensive care unit
IFN-γ: interferon gamma
IL: interleukin
IFN-γ: interferon gamma
IFAD: international fluid academy day
MCP-1: monocyte chemotactic protein-1
MODS: multiple organ dysfunction score
PAMPs: pathogen-associated molecular patterns
PMX-HP: polymyxin B haemoperfusion
PCT: procalcitonin
PRCT: prospective randomized control trial
RCT: randomized control trial
RRT: renal replacement therapies
SAI: sepsis-associated immunosuppression
TNF-alpha: tumour necrosis factor alpha
